# Intestinal dysbiosis during pregnancy and microbiota-associated impairments in offspring

**DOI:** 10.3389/frmbi.2025.1548650

**Published:** 2025-03-20

**Authors:** Yaa Abu, Sabita Roy

**Affiliations:** 1 Medical Scientist Training Program, University of Miami Miller School of Medicine, Miami, FL, United States; 2 Department of Surgery, University of Miami Miller School of Medicine, Miami, FL, United States

**Keywords:** pregnancy, microbiome, metabolites, intestinal dysbiosis, maternal, offspring

## Abstract

The maternal microbiome is increasingly being recognized as a key determinant in various neonatal health outcomes, including offspring immunity, metabolism, brain function, and behavior. While the oral, vaginal, skin, and gut microbiota are significant contributors to the offspring’s postnatal gut microbial seeding, the composition and diversity of the maternal gut microbiome during pregnancy seems to be critical in shaping neonatal health outcomes, even prior to birth. Growing evidence suggests that the balance among the microbial groups in the gut and their interactions with the host are crucial for health. Dysbiotic communities in pregnancy and early in life may lead to disease processes in offspring, though the specific processes by which maternal gut microbes affect offspring gut microbial development are unknown. Here, we summarize research examining gut microbial shifts during pregnancy, and their effects on the diversity and composition of the infant microbiome and on early health outcomes. We also discuss current theories for how the maternal gastrointestinal (GI) tract influences neonatal seeding, and how probiotics during the perinatal period may affect offspring health outcomes.

## Introduction

Pregnancy is a unique state characterized by substantial immunological, hormonal, and metabolic changes that support fetal growth and development (Costantine, 2014; Moya et al., 2014). For instance, immunological changes during this time promote tolerance and include tight regulation of tolerogenic and proinflammatory immune responses to ensure successful implantation and placentation, as well as restoration of maternal antimicrobial immunity (Arck and Hecher, 2013). Mild systemic inflammation has also been documented in pregnant women (Fink et al., 2019), as well as increased levels of progesterone, estrogen, and thyroid hormone levels (Costantine, 2014; Moya et al., 2014). Additionally, metabolic alterations to accommodate growing energy demands lead to an increase in food intake, weight gain, lipogenesis, elevated fasting blood-glucose levels, insulin resistance, glucose intolerance, and low-grade inflammation, with the body mirroring a metabolic-syndrome-like condition (Costantine, 2014; Moya et al., 2014). These metabolic adaptations are critical for a healthy pregnancy and are thought to influence, and be influenced by, changes in the maternal gut microbiome. The composition and diversity of the microbiome have been shown to reflect and potentially contribute to immunological and metabolic adaptations (Koren et al., 2012; Zhang et al., 2015; Jašarević and Bale, 2019; Berry et al., 2021). However, this relationship is likely bidirectional, as metabolic changes required for pregnancy may also drive shifts in the microbiome, emphasizing the complex interplay between host physiology and microbial communities that warrants further investigation.

There is growing recognition that the gut microbiome during pregnancy is closely related to the health of pregnant women as well as their offspring. An imbalance of gut microbiota, termed dysbiosis, during pregnancy has been attributed to antibiotic use during pregnancy, maternal stress, infection, environmental and drug exposures, and high fat diet (HFD), amongst other contributors (Ma et al., 2014; Gohir et al., 2015; Vuillermin et al., 2017; Nyangahu et al., 2018). Several studies have correlated maternal intestinal dysbiosis during pregnancy with an increased risk of childhood obesity, immunological dysfunction, asthma, and neurological disease/neurodevelopmental abnormalities, amongst other conditions (Ma et al., 2014; Gohir et al., 2015; Vuillermin et al., 2017; Nyangahu et al., 2018). In this regard, modulation of the gut microbiota during pregnancy through probiotic, prebiotic, or symbiotic therapies has been attempted to encourage a more balanced ecosystem (Barthow et al., 2016; Sohn and Underwood, 2017; Baldassarre et al., 2018; Swartwout and Luo, 2018). An estimate of 1.3 to 3.6% of pregnant women in the United States and Canada currently supplement with probiotics (Jarde et al., 2018). However, randomized clinical trials on their efficacy at preventing negative microbiota-driven health impairments in offspring are relatively few.

Despite numerous studies linking maternal dysbiosis to significant adverse maternal and neonatal health outcomes (Dunlop et al., 2015; Zhang et al., 2015; Zhuang et al., 2019), the mechanisms through which maternal microbes mediate these effects remain poorly understood. Elucidating microbial shifts in pregnancy with human subjects is challenging (Berry et al., 2021), due to small sample sizes, population demographics, cross-sectional sampling, interpersonal variation in diet, and concomitant bias in self-reported food intake surveys—all of which are significant confounding variables. Preclinical studies have been informative in describing prenatal and postnatal contributions from microbial metabolites produced in the maternal gut that can cross placental and fetal tissues to drive postnatal development or vertical transmission of microbes during birth and in the perinatal period (De Agüero et al., 2016; Kimura et al., 2020). Here, we review the literature on how intestinal dysbiosis during pregnancy affects the gut microbiota in offspring and influences offspring health outcomes. We further describe how maternal probiotic interventions may change these parameters.

## Alterations in the maternal gut microbiota during pregnancy

The gut microbiota includes the many microorganisms, including viruses, bacteria, and fungi, that exist within the gastrointestinal (GI) tract (Clemente et al., 2012). Together, these microorganisms work in a symbiotic relationship with the body and play a crucial role in overall host homeostasis, particularly through development and maintenance of immune function and modulation of host nutrition and energy metabolism (Clemente et al., 2012). Alterations in the gut microbiota during pregnancy have garnered much attention in recent years. During pregnancy, the body undergoes profound physiological changes, with endocrine, metabolic and immunological adaptations focused on expanding neonatal nutrient and energy demands and promoting maternal-fetal immune tolerance (Costantine, 2014; Moya et al., 2014).

**Figure 1:**
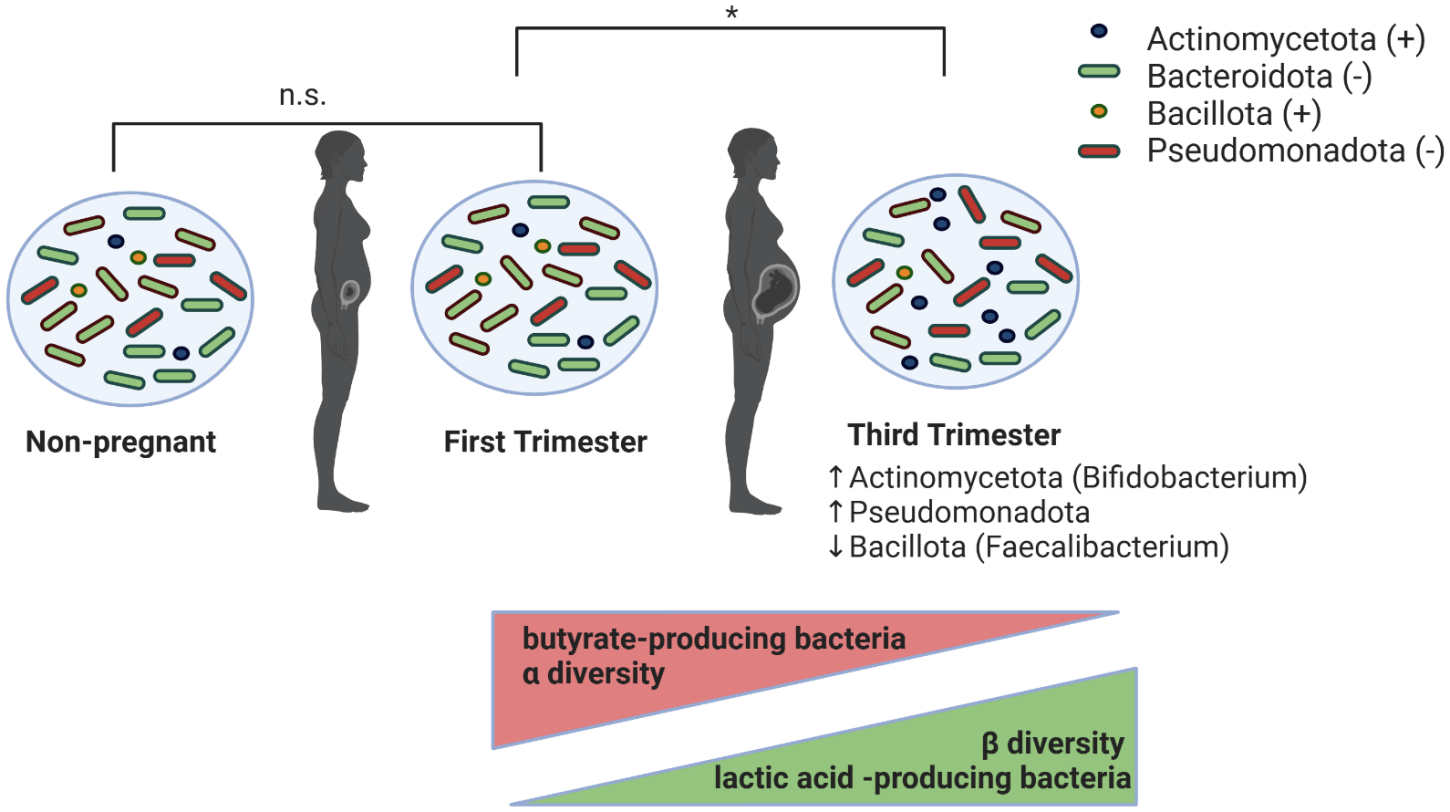
Proposed changes in maternal intestinal microbiota during pregnancy. Gut microbiota in first-trimester pregnant women are indistinguishable (denoted by n.s. in figure) from those in non-pregnant women. From the first to the third trimester of pregnancy, the maternal gut microbiota undergoes significant changes (denoted by * in figure), marked by increases in Actinomycetota and Pseudomonadota and decreases in Bacillota phyla. Additionally, during this period of time, there is a decrease in α-diversity and butyrate-producing bacteria, and an increase in β-diversity and lactic acid-producing bacteria.

In the last decade, the rise of omics- and next generation sequencing technology have allowed for analysis of the temporal variation in the maternal gut microbiota during pregnancy. Some have revealed profound alterations in gut microbiota (**Figure 1**) that may describe yet another physiological shift during pregnancy (Collado et al., 2008; Ferretti et al., 2018), while others have found a relatively stable gut microbial profile during pregnancy (DiGiulio et al., 2015; Goltsman et al., 2018; Yang et al., 2020). The first trimester of pregnancy has consistently been shown to resemble that of healthy, non-pregnant women, whose gut microbiomes show a predominance of Bacillota, particularly *Clostridiales* (Nuriel-Ohayon et al., 2016; Edwards, 2017; Mesa et al., 2020). However, studies vary on their reporting of gut microbial composition following the first trimester. In a study of 91 pregnant women in Finland, Koren et al., showed dramatic remodeling of the gut microbiota over the course of pregnancy using clinical data, stool samples collected during each trimester, and self-reported diet information. They reported significant microbial remodeling, including an enrichment of Actinomycetota and Pseudomonadota—taxa involved in local inflammatory modulation—and a decrease in *Faecalibacterium* during the third trimester (Koren et al., 2012). Third-trimester samples also exhibited lower individual richness (α-diversity) but greater between-subject diversity (β-diversity), which correlated with weight gain, insulin sensitivity, and increased fecal cytokines (Koren et al., 2012). Notably, fecal microbial transplantation (FMT) of third trimester feces to germ-free mice resulted in greater adiposity and insulin insensitivity compared to transfer of first trimester feces (Koren et al., 2012). While these findings suggest a shift resembling obesity-associated metabolic inflammation, they likely reflect an adaptive physiological response rather than pathological dysbiosis. Unlike the obesity-associated microbiome, which enhances energy extraction, the third-trimester microbiome was linked to increased energy loss in stool, highlighting distinct host-microbe interactions (Koren et al., 2012). Furthermore, Pseudomonadota enrichment, often associated with inflammation, appears to be a regulated adaptation rather than a marker of disease (Koren et al., 2012).

Other studies have reported a relatively stable gut microbiota across pregnancy. A 16S ribosomal RNA (rRNA) gene survey of weekly variation in the vaginal, gut, and oral microbiota from 49 women during pregnancy demonstrated relative stability over time at each body site during pregnancy (DiGiulio et al., 2015). Similarly, a 16S rRNA gene survey of fecal samples from 1479 pregnant women identified a core microbiota of pregnant women, which displayed a similar overall structure to that of age-matched non-pregnant women (Yang et al., 2020). Specifically, the overall gut microbial structure, characterized by enterotypes, dominant taxonomic, and functional composition of pregnant women was similar to that of age-matched non-pregnant women (Yang et al., 2020). This population-level survey suggested that gestational age-associated variation in the gut microbiota, from the ninth week of gestation to antepartum, is relatively limited, with individual heterogeneity, rather, as the main force shaping the gut microbiome during pregnancy (Yang et al., 2020). Additionally, using whole-community shotgun sequencing of human microbiome samples from three body sites (vagina, oral cavity, and gut) in 10 pregnant subjects, Goltsman et al., observed that while the individual was the strongest source of sample-sample-variation, a significant gestational age (time) effect was observed for most subjects at all body sites (Goltsman et al., 2018).

The inconsistent observations of these aforementioned clinical studies highlight the inherent difficulties and complexities of patient sampling. A range of factors including physical conditions before pregnancy (pre-pregnancy weight, hormone levels), psychological and environmental factors, dietary patterns, and patient demographics (e.g., age, ethnicity, geographical location) influence the gut microbiota during pregnancy, but are inconsistently accounted for amongst studies (Yang et al., 2020). The field is still lacking detailed longitudinal analyses using larger patient cohorts that extend past examinations of phylogenetic composition but also incorporate metagenomic analyses. A multi-omic approach incorporating analysis of the microbiome, metabolome, and proteome will provide a comprehensive overview of dynamics in the gut microbiome, including metabolic and functional activities that exist during pregnancy.

## Offspring postnatal gut microbial development

**Figure 2:**
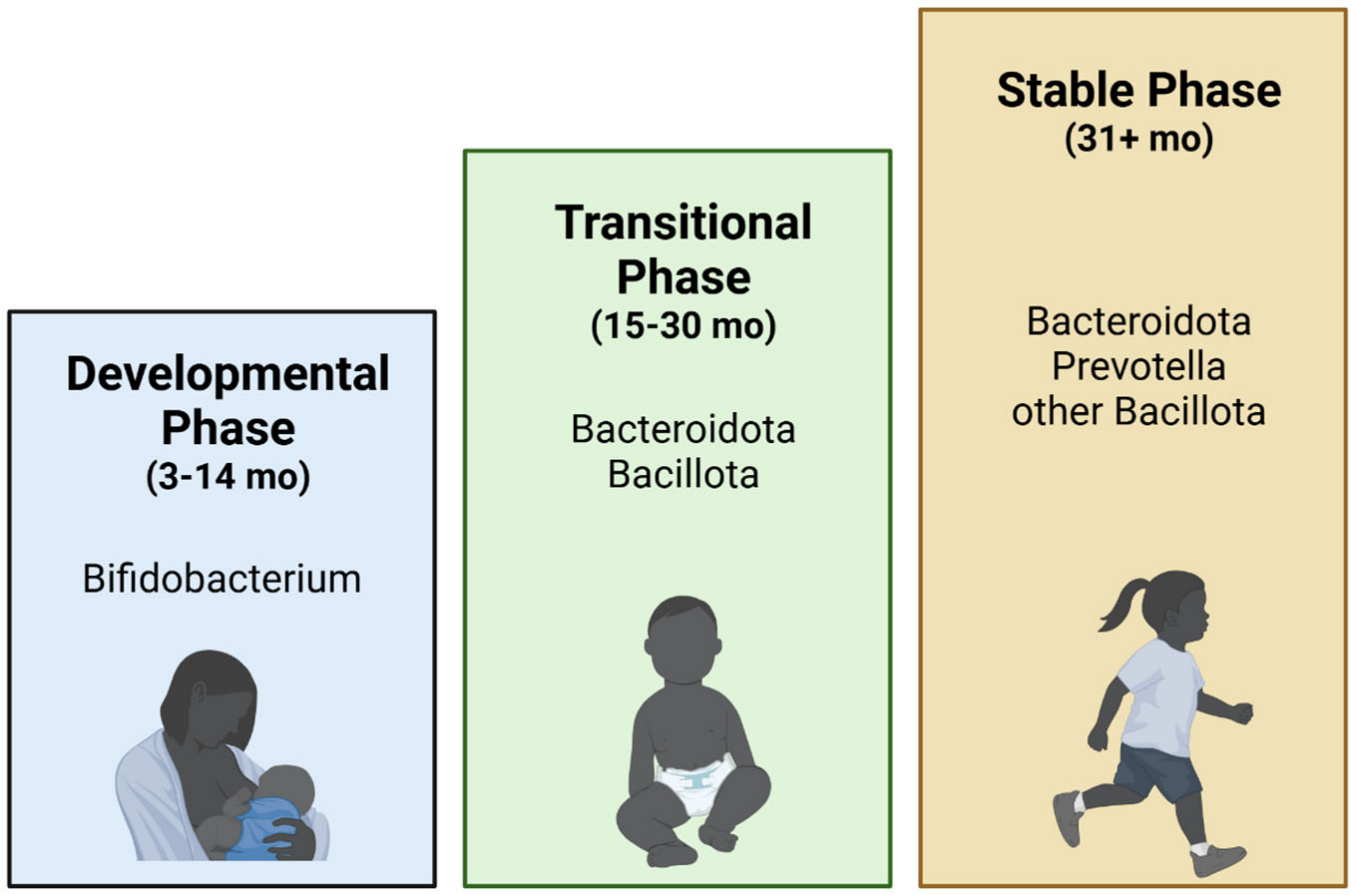
Infant gut microbiome development. Infant human gut microbiome maturation is characterized by three stages (Stewart et al., 2018): the 1) Developmental (3–14 months), dominated by *Bifidobacterium*; 2) Transitional (15–30 months), dominated by Bacteroidota and Bacillota; and 3) Stable (31+ months), dominated by Bacteroidota, *Prevotella*, and other Bacillota. The Developmental and Transitional stages are the most dynamic stages of gut microbiome development, while relatively minor shifts in the gut microbiome are observed in the Stable phase (Stewart et al., 2018).

Offspring gut microbial development is determined by maternal-offspring exchange of microbiota, which predominately has been shown to start at birth upon exposure to vaginal, fecal, and skin microbiota (Mueller et al., 2015; Hill et al., 2017; Tanaka and Nakayama, 2017; Dalby and Hall, 2020; Wernroth et al., 2022). Microbiome seeding is affected by several practices, including gestational age, mode of delivery, birth weight, use of antibiotics, breastmilk vs formula feeding, timing of the introduction of solid foods, and cessation of milk feeding (Mueller et al., 2015; Hill et al., 2017; Tanaka and Nakayama, 2017; Dalby and Hall, 2020; Wernroth et al., 2022). Amongst these, birth mode, perinatal antibiotics, and formula feeding have been shown to be major determinants in diversity and colonization patterns (Mueller et al., 2015; Hill et al., 2017; Tanaka and Nakayama, 2017; Dalby and Hall, 2020; Wernroth et al., 2022). Following birth, the infant gut microbiome development is characterized by three phases (**Figure 2**) (Stewart et al., 2018): 1) the developmental phase (months 3–14), 2) the transitional phase (months 15–30), and 3) the stable phase (months 31–46). The developmental and transitional phases are dynamic periods of infant gut microbial development, with the establishment of “adult-like” microbiota during the stable phase of development around 2.5-3 years of age (Mueller et al., 2015; Hill et al., 2017; Tanaka and Nakayama, 2017; Dalby and Hall, 2020; Wernroth et al., 2022). The developmental phase occurs shortly after birth during lactation and results in dominance of *Bifidobacterium* (Tanaka and Nakayama, 2017). The breastmilk microbiota is dominated by genera *Staphylococcus, Streptococcus, Serratia, Pseudomonas*, *Corynebacterium, Ralstonia*, *Propionibacterium, Sphingomonas*, and *Bradyrhizobiaceae*, as well as *Bifidobacterium* and *Lactobacillus* spp (Mueller et al., 2015). Interestingly, specific species and strains of *Bifidobacterium* have evolved to selectively digest human milk oligosaccharides (HMOs) in breastmilk, thereby producing various microbial fermentation products (such as the short-chain fatty acid (SCFA) acetate), and metabolizing breast milk amino acids into aromatic lactic acid, which are critical for maintaining the infant gut epithelium and promoting an acidic environment in the gut (Dalby and Hall, 2020). The next stage, the transitional phase, is marked by weaning from human milk in addition to the introduction of solid foods (Mueller et al., 2015; Hill et al., 2017; Tanaka and Nakayama, 2017; Dalby and Hall, 2020; Wernroth et al., 2022). This promotes the growth of Bacteroidota and Bacillota as well as anaerobic organisms involved in the utilization of solid foods (Tanaka and Nakayama, 2017). Last, the stable phase, achieved at approximately 3 years of age, results in the establishment of an adult-type complex microbiome dominated by the phyla Bacteroidota, and Bacillota (Tanaka and Nakayama, 2017). While immune and metabolic programming occur throughout the developmental and weaning stages, they continue to be shaped during this phase with these intestinal microbiota further contributing to immune system maturation, metabolic regulation, and host homeostasis through interactions along the gut-brain-axis (Mueller et al., 2015; Hill et al., 2017; Tanaka and Nakayama, 2017; Dalby and Hall, 2020; Wernroth et al., 2022). Accordingly, disruption of neonatal gut microbial assembly is associated with disease in later life (Mueller et al., 2015; Hill et al., 2017; Tanaka and Nakayama, 2017; Dalby and Hall, 2020; Wernroth et al., 2022).

## Evidence of the maternal gut microbiota as a major donor of infant-acquired strains

The maternal gut microbiome is widely accepted as playing a crucial role in the seeding of the neonatal gut microbiome. However, the transmission routes of the infant pioneering microbiome are poorly understood. While a large body of literature supports the observation that the maternal gut microbiota can be vertically transmitted to infants, direct evidence in the majority of these studies is lacking. Much of what is known about vertical transmission of microbes from women to their infants has been obtained using cultivation-based (Makino et al., 2011) or taxonomic profiling limited to the genus and/or species level (Jost et al., 2014; Milani et al., 2015; Duranti et al., 2017; Fehr et al., 2020). However, many vertically transmitted microorganisms are difficult to cultivate, limiting interpretation of the true extent of microbial transmission by cultivation methods alone. Additionally, as many microbial species are common among unrelated individuals (Bäckhed et al., 2015) and there is great overlap between microbiota normally present in the gut, vagina, and breast milk (Wang et al., 2020), inferring vertical transmission at lower taxonomic resolution even at the species level is insufficient. Even in cases where a species is identified in both mother and infant, it still remains inconclusive if this is due to vertical transmission, as species exhibit considerable strain heterogeneity (Bäckhed et al., 2015).

Recent advances in metagenomic technology have allowed for strain-resolved computational profiling to characterize the transfer of microbes from women to their infants in culture-free environmental samples and complex mixtures of bacteria (Yassour et al., 2018). Shotgun metagenomics and strain-level profiling is becoming a gold standard for the identification of vertical transmission events, as this more effectively can discriminate whether shared microbes are of the same genetic variant based on the species core genome and non-core genome (Yassour et al., 2018). Furthermore, by combining metagenomic sequencing with metatranscriptomics, the transcriptional activity of these vertically transmitted microbes can be characterized to ascertain transient or longstanding colonization of the neonatal intestine (Asnicar et al., 2017). Still, even with these metagenomic technologies, validation with integrative functional analyses including laboratory cultivation, isolation, and biochemical characterization of specific strains is necessary (Zhong et al., 2022).

A small but growing body of work is now providing direct evidence of maternally transmitted gut microbes using metagenomic technology at strain-specific resolution (Makino et al., 2011; Milani et al., 2015; Nayfach et al., 2016; Asnicar et al., 2017; Duranti et al., 2017; Ferretti et al., 2018; Makino, 2018; Yassour et al., 2018; Zhong et al., 2022). In a longitudinal study of 25 mother/infant pairs across multiple body sites, Ferretti et al., concluded that of the maternal sources of transmission (tongue dorsum, skin, vagina, stool), the gut microbiome was the largest donor of the infant-acquired strains, with maternal gut strains more persistent in the infant gut and ecologically better adapted than those acquired from other sources (Ferretti et al., 2018). Maternal routes of transmission were confirmed by single nucleotide variant (SNV) identity patterns (e.g., species belonging to *Bifidobacteria* and *Bacteroides*) or pangenome analysis due to genome plasticity and a large set of accessory genes (e.g., *Escherichia coli*). Similarly, in a cohort of five mother/infant pairs, Asnicar et al., detected several species, including *Bifidobacteria* typical of the infant gut but also *Clostridiales* species usually found in the adult intestine, that had substantial genetic diversity between different pairs but identical genetic profiles between mother/infant pairs, indicative of vertical transmission (Asnicar et al., 2017). Additionally, their metatranscriptomics analysis allowed for the study of *in vivo* gene expression of vertically transmitted microbes, where they found that transmitted strains of *Bacteroides* and *Bifidobacterium* species were transcriptionally active in the guts of both adult and infant (Asnicar et al., 2017). While the aforementioned studies examined transmission of maternal dominant GI strains to offspring, Yassour et al., further conducted longitudinal metagenomic sequencing from 44 mother/child pairs and identified transmission events on both dominant as well as secondary strains from women to neonates to identify gene signatures to explain different inheritance patterns. In some instances, a mother’s secondary strain was transmitted to neonates rather than the dominant, as in the case of *Bacteroides uniformis*, and it was hypothesized that the dominant strains in some women lacked specific genes that may confer a selective advantage in the infant gut. By examining families with evidence of secondary strain transmission, it was found that in cases where an infant was colonized by a mother’s secondary strain of *B. uniformis*, the maternal dominant strain often lacked a specific starch utilization system (Sus) involved in processing complex glycans by a cell envelope-associated multiprotein system (Martens et al., 2009). Notably, the infant gut utilizes human milk oligosaccharides found in the mother’s breast milk that act as prebiotics for commensal bacteria, suggesting that the existence of certain Sus genes such as SusC in the infant gut may confer a selective advantage for glycan metabolism (Martens et al., 2009).

Aside from the mother vertically transmitting microbes directly to neonates, it has now been shown that bacteria not transmitted from mother to baby can still influence the neonatal microbiome through maternal transmission of specific genes from one bacterium to other bacteria of different species in the neonate termed horizontal gene transfer (Vatanen et al., 2022). This provides a new indirect mechanism complementing the transmission of specific bacterial strains from mother to infant by characterizing interspecies transfer of mobile genetic elements between maternal and infant microbiomes. Vatanen et al., tracked the co-development of microbiomes and metabolomes in 74 infants and 137 women from late pregnancy to 1 year of age. Besides functions related to mobile genetic elements, transmitted genes encoded functions related to genes for metabolic pathways for food digestion including carbohydrate utilization, amino acid metabolism, and iron acquisition and storage. In particular, *Bacteriodes cellulosilyticus* was found to be a major donor species in gene sharing events and was positively associated with HMO-metabolizing glycoside hydrolases and species that scavenge liberated HMO glycans in infant samples (Vatanen et al., 2022). Such observations highlight the importance of metabolites produced by the maternal microbiota in neonatal microbiota seeding independent of the transfer of microbial strains.

Together, these studies provide more direct insight into mother-to-child bacterial transmission events and expand our understanding of early colonization of the infant gut. However, increasing cohort size and expanding analyses to include other potential maternal/infant body sites and environmental settings will be necessary to better illustrate seeding and development of the neonatal gut microbiome.

## Intestinal dysbiosis during pregnancy and its reported effects on offspring

**Figure 3:**
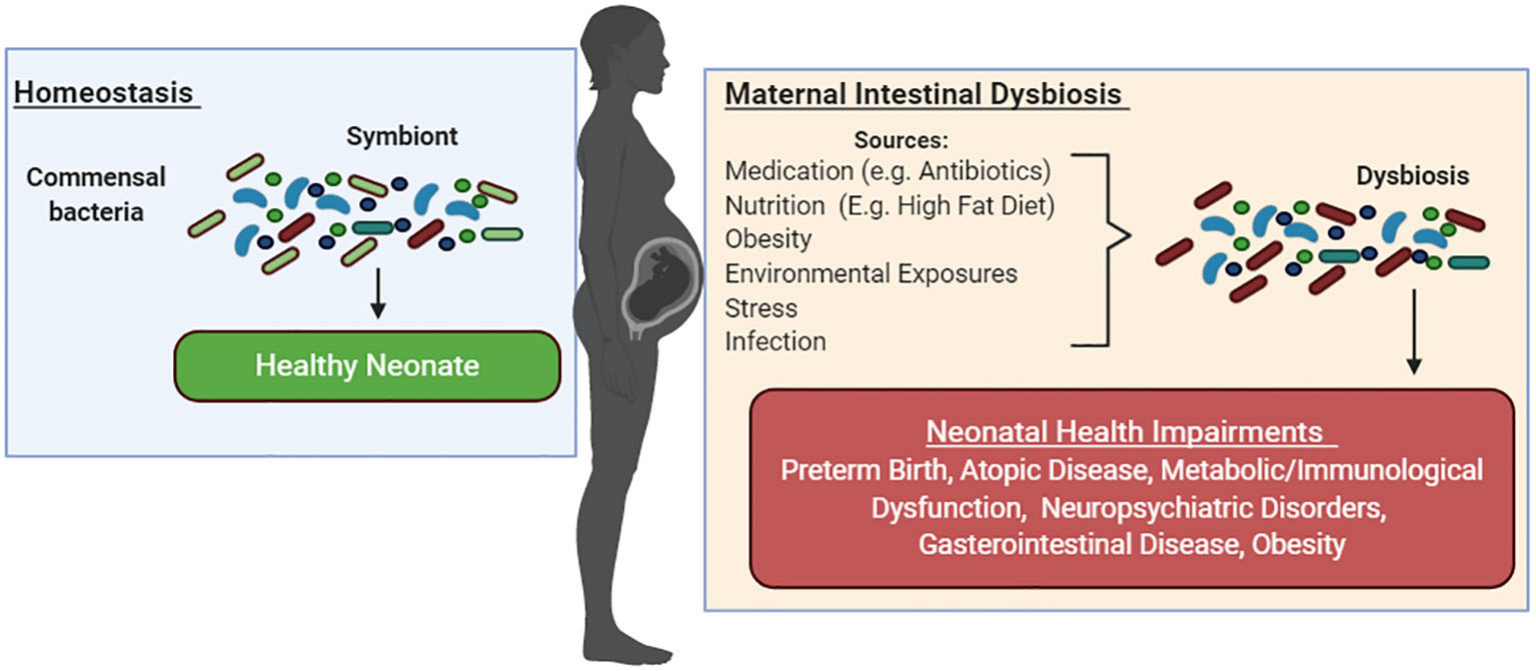
Maternal intestinal dysbiosis during pregnancy negatively shapes infant health. Gut microbiota symbiosis has been associated with healthy pregnancies. Medication use (e.g., antibiotics), poor nutrition (e.g., HFD), maternal obesity, environmental exposures, stress, and infections during pregnancy have been linked to maternal dysbiosis during pregnancy, alterations in neonatal gut microbiota composition, and adverse health outcomes. Among these, preterm birth, atopic diseases, metabolic/immunological dysfunction, neuropsychiatric disorders, GI disease, and obesity have been reported in infants and children born to mothers with intestinal dysbiosis during pregnancy in both clinical and preclinical studies.

With the inherent variability in clinical studies, preclinical models have largely been able to inform of changes in gut microbial composition and diversity during pregnancy and the ensuing effects in offspring. Together, these studies have shown associations between intestinal dysbiosis during pregnancy from medication use (e.g., antibiotics), poor nutrition (e.g., HFD), maternal obesity, environmental exposures, stress, and infections; alterations in neonatal gut microbiota composition; and adverse health outcomes. Among these, atopic diseases (McKeever et al., 2002; Wood et al., 2013; Örtqvist et al., 2014; Timm et al., 2017; Loewen et al., 2018; Al Nabhani et al., 2019; Alhasan et al., 2020; Yuan et al., 2023), metabolic/immunological dysfunction (De Agüero et al., 2016; Gonzalez-Perez et al., 2016; Bruce-Keller et al., 2017; Xie et al., 2018; Örtqvist et al., 2019; Nyangahu et al., 2020), neuropsychiatric disorders (Buffington et al., 2016; Tochitani et al., 2016; Bruce-Keller et al., 2017), and GI disease (Miyoshi et al., 2017a; Schulfer et al., 2018; Xie et al., 2018; Örtqvist et al., 2019) have commonly been reported in infants and children born to mothers with intestinal dysbiosis during pregnancy in both clinical and preclinical studies (**Figure 3**).

### Antibiotics

Antibiotics are the most commonly prescribed drug during pregnancy (Codagnone et al., 2019). In Western countries, they are used in 30–40% of deliveries, mainly during the peripartum period to treat chorioamnionitis and early-onset sepsis, prevent preterm birth, manage maternal infections after cesarean section, and prevent neonatal Group B Streptococcus infection in colonized mothers (Moya-Alvarez and Sansonetti, 2022). Repeat epidemiological and preclinical studies have shown that antibiotics administered during pregnancy decrease bacterial load and significantly alter the β-diversity in fecal samples. Offspring have consistently mirrored these trends in gut microbiome α- and β-diversity and differentially enriched species. These studies have additionally correlated prenatal and/or antibiotic exposure early in life with a range of adverse outcomes in offspring, including alterations in systolic BP (Galla et al., 2020), body weight (Tochitani et al., 2016; Miyoshi et al., 2017a; Isaevska et al., 2020), metabolic profiles (Tochitani et al., 2016; Klancic et al., 2020; Lamont et al., 2020), immune response/mortality following infection (Gonzalez-Perez et al., 2016; Nyangahu et al., 2018), atopic disease (Stensballe et al., 2013; Metzler et al., 2019; Alhasan et al., 2020), rate of spontaneous colitis/inflammatory bowel disease (Miyoshi et al., 2017a; Schulfer et al., 2018; Örtqvist et al., 2019), intestinal permeability (Fåk et al., 2007), behavior (Tochitani et al., 2016), and bacterial metabolite production (Arrieta et al., 2015; Tulstrup et al., 2018; Alhasan et al., 2020).

Clinical studies are inherently limited in their ability to establish a causal link between maternal antibiotic exposure, neonatal gut dysbiosis, and adverse effects. However, preclinical studies, especially those conducting FMT of antibiotic-perturbed feces into pregnant germ-free mice, strongly suggest that dysbiosis early in life may be a key mediator in the development of disease later in life. In a hallmark paper, it was found that embryos from antibiotic-treated and germ-free dams had reduced expression of genes related to axonogenesis in the brain, and exhibited deficient thalamocortical axons and impaired outgrowth of thalamic axons in response to cell-extrinsic factors (Vuong et al., 2020). Furthermore, adult offspring displayed disrupted neurobehavioral responses to forepaw and hindpaw tactile stimuli (Vuong et al., 2020). Gnotobiotic colonization of microbiome-depleted dams with a limited consortium of bacteria rescued abnormalities in fetal brain gene expression and thalamocortical axonogenesis (Vuong et al., 2020). Additionally, maternal supplementation with select microbiota-dependent metabolites abrogated deficiencies in fetal thalamocortical axons, and were linked to improvement in forepaw and hindpaw tactile sensitivity in two aversive somatosensory behavioral tasks in adult offspring (Vuong et al., 2020).

Other studies have provided further evidence that antibiotic-induced maternal dysbiosis during a critical window of gut microbial development and immune programming can have lasting effects on offspring using susceptible animal models. For instance, in the interleukin (IL)-10 knockout (KO) mouse model, genetic risk in IL-10 KO mice is not sufficient to cause IBD if raised germ-free or housed in a Helicobacter hepaticus-free environment (Sellon et al., 1998). Another study examined the temporal impact of the broad-spectrum antibiotic cefoperazone administered during the peripartum period on both the maternal and offspring microbiota and found that offspring from antibiotic-exposed dams developed dysbiosis that persisted until adulthood (Miyoshi et al., 2017b). This was associated with aberrant immune development and susceptibility of offspring to the development of spontaneous and chemically (dextran sodium sulfate)-induced colitis in IL-10 KO mice (Miyoshi et al., 2017b). FMT of antibiotic-exposed dams’ microbiota into germ-free IL-10 KO mice resulted in a similar skewing of the host immune response in recipients, suggesting a causal link between gut dysbiosis and colitis and pro-inflammatory immune development in genetically susceptible offspring (Miyoshi et al., 2017b). Similar findings were shown by Schulfer et al., in which germ-free pregnant mice inoculated with feces from antibiotic-treated mice transmitted their perturbed microbiota to their offspring, which remained distinct from controls for at least 21 weeks (Schulfer et al., 2018). The offspring of IL-10 KO germ-free mice receiving perturbed microbiota during pregnancy had markedly increased susceptibility to colitis (Schulfer et al., 2018).

Additionally, preclinical studies have found that gestational antibiotic exposure increases susceptibility to asthma in offspring, which is associated with dysbiosis and decreased levels of SCFA in offspring (Alhasan et al., 2020). This corroborates human findings that infants at risk of asthma exhibit transient gut microbial dysbiosis during the first 100 days of life, which is accompanied by reduced levels of fecal acetate (a SCFA) and dysregulation of enterohepatic metabolites (Arrieta et al., 2015). Interestingly, inoculation of *Lachnospira, Veillonella, Faecalibacterium*, and *Rothia*, which were significantly decreased in children at risk of asthma, into germ-free pregnant mice ameliorated airway inflammation in their adult progeny, demonstrating a causal role of these bacterial taxa in averting asthma development (Arrieta et al., 2015). Similarly, high fiber or acetate feeding led to marked suppression of allergic airways disease, a model for human asthma, by enhancing T regulatory cell numbers and function and increasing acetylation at the Foxp3 promoter (Thorburn et al., 2015).

Other studies have used cross-fostering strategies in which cross-fostering prenatally-antibiotic exposed offspring to control mothers on postnatal day 1 significantly improved birth weight, spontaneous behavior in both familiar and unfamiliar environments, and spatial preference for locomotion (Tochitani et al., 2016). Conversely, naïve offspring who were cross fostered to antibiotic-treated dams exhibited similar phenotypes as prenatally antibiotic-exposed offspring (Tochitani et al., 2016). Gestational antibiotic treatment was hypothesized to cause microbiome-mediated alterations in breastmilk composition postnatally given the profound role of the microbiome in host-nutrient metabolism, which subsequently could affect offspring development and phenotypic responses (Tochitani et al., 2016). Collectively, these observations support findings in human subjects that peripartum antibiotic-induced dysbiosis is transmitted to offspring and establishes a link between maternal and neonatal dysbiosis and the development of disease in at-risk individuals.

### Environmental exposures/drugs of abuse

Extensive knowledge about the harmful effects of substance abuse during pregnancy on the developing fetus exist. For instance, alcohol exposure during pregnancy leads to lifelong physical, behavioral, and cognitive disabilities described as fetal alcohol spectrum disorder (Ehrhart et al., 2019). Additionally, opioid use during pregnancy is associated with neonatal opioid withdrawal syndrome (NOWS), which poses a serious threat to newborns and is characterized by a diverse constellation of symptoms including respiratory distress, tremors, emesis, seizures, irritability, feeding difficulties, and hypertonia after birth (Ross et al., 2015; McQueen and Murphy-Oikonen, 2016). Still, use of these agents is reported during pregnancy, with polysubstance use during pregnancy of growing concern. A 2017 study found that more than half of pregnant women who used opioids for nonmedical reasons reported drinking more than five drinks with alcohol per day during a 1-month period of time (Jarlenski et al., 2017).

Notably, the impact of several substances of abuse on the microbiome have been extensively studied, though mainly in adult populations. Though human and preclinical models have shown considerable variability in describing the microbial composition with opioid use, both have consistently pointed towards microbial dysbiosis with opioid use in adults (reviewed in Jalodia et al., 2022). Similarly, alcohol consumption is known to affect GI mucosal integrity and gut microbiota composition (Mutlu et al., 2009; Yan et al., 2011; Peterson et al., 2017). Still, phylogenetic and metagenomic analyses in offspring prenatally exposed to substances of abuse overall are lacking. With regards to prenatal opioid exposure, recent reports have shown that prenatally opioid-exposed offspring exhibit lasting alterations in their gut microbiome composition that similarly reflect loss of commensal bacteria and expansion of pathogenic flora as in adult models of opioid exposure (Abu et al., 2021; Antoine et al., 2022; Grecco et al., 2022; Lyu et al., 2022; Abu et al., 2024). Both brief (Abu et al., 2021) and chronic opioid exposure (Grecco et al., 2021; Antoine et al., 2022; Lyu et al., 2022; Abu et al., 2024) have been associated with maternal and neonatal microbial dysbiosis. While studies differ on their reports of α-diversity (Abu et al., 2021; Grecco et al., 2021; Antoine et al., 2022; Lyu et al., 2022; Abu et al., 2024), all have documented profound changes in microbial composition apparent at weaning (Abu et al., 2021; Grecco et al., 2021), adolescence (Antoine et al., 2022), or adulthood (Antoine et al., 2022; Lyu et al., 2022). Interestingly, one report further showed that hypersensitivity to thermal and mechanical pain in prenatally opioid exposed offspring may be mediated by the gut microbiome by using FMT and probiotics to alter maternal and/or offspring gut microbiome composition (Abu et al., 2024). Taxonomical analysis revealed that prenatal methadone exposure resulted in significant alterations in fecal gut microbiota composition, including depletion of *Lactobacillus*, *Bifidobacterium*, and *Lachnospiracea* sp and increased relative abundance of *Akkermansia, Clostridium sensu stricto 1*, and *Lachnoclostridium* (Abu et al., 2024). Supplementation of the probiotic VSL#3 in dams altered offspring gut microbial composition and rescued hypersensitivity to thermal and mechanical pain in prenatally methadone-exposed offspring (Abu et al., 2024). In regard to prenatal exposure to alcohol, further studies also demonstrate strong associations between maternal and offspring gut dysbiosis. In a cohort of 29 mother-child dyads, in which 10 mothers reported alcohol consumption during pregnancy, it was found that both dams and offspring prenatally exposed to alcohol exhibited the same trend in α- and β-diversity; specifically, increased α-diversity in alcohol consumption groups and significant alterations in the gut microbiome of both dams and offspring exposed to alcohol was observed (Wang Y. et al., 2021). Maternal alcohol consumption was positively correlated with *Phascolarctobacterium* and *Blautia* and negatively correlated with *Faecalibacterium* (Wang Y. et al., 2021), consistent with other studies (Yan et al., 2011). In newborns, enrichment of *Megamonas* was found which positively correlated with maternal alcohol consumption (Wang Y. et al., 2021).

Still, much work is needed on how other commonly used substances during pregnancy, including nicotine and marijuana, affect the maternal and neonatal gut microbiomes. Additionally, the combined effect of polysubstance use on the maternal and neonatal microbiomes remains to be examined. Outside of drugs of abuse, very few studies have shown how other environmental exposures during pregnancy affect the developing offspring. For instance, it has been shown that triclocarban exposure in rats can reduce α-diversity in dams and significantly alter β-diversity in both dams and offspring; this was further associated with enlarged abdomens, diarrhea, and abnormal GI histology in offspring (Kennedy et al., 2016). This calls for more comprehensive investigations probing the effect of substances of abuse and environmental exposures on the maternal and neonatal gut microbiomes.

### Infection

Maternal infections during pregnancy can disrupt the maternal gut microbiome, leading to downstream effects on offspring microbiota, immunity, and health outcomes. These interactions vary widely depending on the type of infection and its impact on maternal-infant microbial and immune dynamics. Interestingly, it has been shown that maternal helminth infection can protect offspring from HFD-induced obesity through gut microbiota and SCFA alterations (Su et al., 2023). SCFA supplementation to pups of uninfected control mothers mitigated HFD-induced weight gain, which corresponded with changes in gut bacterial colonization (Su et al., 2023). Notably, recent reports indicate that even cleared or undetectable infections may have a long-lasting impact on immune development in offspring. In one report, while host treatment with ivermectin cleared preconception helminth infection, maternal stool during pregnancy and breastmilk postnatally were still found to be significantly altered (Nyangahu et al., 2020). Offspring of dams with preconception helminth infection had a significantly decreased abundance of *Clostridiaceae* and an increased abundance of *Erysipelotrichaceae* and *Coriobacteriaceae* (Nyangahu et al., 2020). Additionally, changes in the neonatal microbiome were associated with increased frequency and activation of B cells and CD4 T cells in spleens (Nyangahu et al., 2020). Williamson et al., further mirror that perinatal exposure to helminths may have protective effects against persistent immune sensitization and cognitive dysfunction induced by early-life infection (Williamson et al., 2016). Additionally, helminth infection prevented the shift in genera within the Actinomycetota and Mycoplasmatota phyla to genera in the Bacteroidota phylum in neonatal *E.coli*-infected rats (Williamson et al., 2016).

Bender et al., similarly show that HIV infection during pregnancy has a lasting impact on the neonatal microbial composition of their HIV-exposed, uninfected infants. Improvements in antiretroviral therapy (ART) have led to a growing population of HIV-exposed, uninfected infants who have been shown to experience higher morbidity and twice the mortality of controls in the same community (Chilongozi et al., 2008; Slogrove et al., 2012; von Mollendorf et al., 2015). Surprisingly, though very few differences were observed in the microbiomes (vagina, breast milk, areolar skin) of HIV-infected mothers compared to controls, maternal HIV infection was associated with significant changes in the microbiome of HIV-exposed, uninfected infants (Bender et al., 2016). Specifically, these infants exhibited lower α-diversity, with a positive relationship between α-diversity and CD4 count or viral load (Bender et al., 2016). They were also found to have more abundant populations of *Pseudomonadaceae*, which were found to be most predictive of maternal HIV status, and *Thermaceae* in their stool compared to control infants (Bender et al., 2016). Differences in maternal HMO composition were also observed in lactating mothers, which was predicted to direct growth of specific microbiota or have downstream prebiotic effects on the growth and colonization of other bacterial species in the infant microbiome (Bender et al., 2016).

In regard to respiratory infections, maternal *Lactobacillus johnsonii* supplementation was found to alter both the maternal and neonatal gut microbiome which regulated immunity to and enhanced airway protection against RSV infection in offspring; this was independent of changes in viral clearance as gene expression of RSV F protein was unchanged between control and experimental groups (Fonseca et al., 2021). Importantly, the COVID-19 pandemic has now produced a growing population of neonates pre-exposed to SARS-CoV-2 *in utero*. It remains largely undetermined how gestational SARS-CoV-2 affects the maternal and neonatal gut microbiomes, and whether there are lasting effects in neonates. Recent studies have demonstrated that SARS-CoV-2 can infect and replicate in enterocytes of the human small intestine, and that the activity of the angiotensin-converting enzyme receptor 2 (ACE2) is influenced by and affects GI function (Juárez-Castelán et al., 2022). Growing studies are now showing altered gut microbiota in SARS-CoV-2 positive patients (Zuo et al., 2020a; Zuo et al., 2020b; Yeoh et al., 2021; Zuo et al., 2021; Juárez-Castelán et al., 2022). Reduction of *Bifidobacteria*, *Faecalibacterium prausnitzii*, or *Eubacterium rectale* has been reported in COVID-19 patients which correlated with disease severity and dysfunctional immune responses (e.g., elevations in C reactive protein, lactate dehydrogenase, aspartate aminotransferase and gamma-glutamyl transferase) (Zuo et al., 2020a; Zuo et al., 2020b; Yeoh et al., 2021; Zuo et al., 2021). Studies examining gut microbiome composition in maternal-infant pairs are few. Interestingly, one study detected SARS-CoV-2 RNAs or spike protein in the stool of 11 out of 14 preterm SARS-CoV-2 negative newborns (per nasal swab) born to mothers with resolved COVID-19 weeks prior to delivery (Jin et al., 2022), which may suggest persistent viral reservoirs in the intestines of newborns. However, the studies referenced were conducted during the early stages of the COVID-19 pandemic and may represent preliminary investigations. Given the dynamic nature of the pandemic and the evolving understanding of its impacts, it is essential to recognize that these studies offer limited insights.

As a whole, these studies contribute additional evidence regarding the potential impact of disruptions in the maternal and neonatal microbiomes on offspring immunity, even with mothers who may be well-nourished and parasitically/virologically suppressed during pregnancy. Future studies will need to further investigate contributions from the microbiome and their byproducts at the maternal-fetal interface, the placenta, or postpartum via breastfeeding to gain further insight into gestational microbial shaping of offspring postnatal outcomes.

### Diet/lifestyle

The effect of diet and lifestyle on the maternal and neonatal microbiomes has been the subject of multiple investigations. Gut microbial composition is primarily driven by diet, which provides selective metabolic pressures promoting or hindering the growth of certain microbiota through substrate availability. Colonization of germ-free mice with an obese microbiota increases their total body fat relative to those colonized with a lean microbiota, suggesting a causal link between the gut microbiota and obesity (Turnbaugh et al., 2006). The rise of the Western Diet, composed of highly refined carbohydrate and fats but low fermentable fiber content, is strongly implicated in the growing prevalence of type 2 diabetes mellitus, obesity, and other metabolic disease (Di Gesù et al., 2022). Individuals with obesity typically exhibit an increased relative abundance of Bacillota and decreased abundance of Bacteroidota, which may skew towards obesity by altering energy procurement from food (Ley et al., 2006; Clarke et al., 2012; Magne et al., 2020). This is recapitulated in pregnant individuals and preclinical models of HFD or obesity during pregnancy (Urbonaite et al., 2022). Pregnant individuals with overweight or obesity display shifts in gut microbiota, including increased Bacillota (*Clostridium, Staphylococcus)* and *Enterobacteriaceae*, along with decreased Bacteroidota and the commensal *Bifidobacterium* (Collado et al., 2008; Santacruz et al., 2010; Zacarías et al., 2018; Urbonaite et al., 2022). Additionally, in pregnant women with overweight or obesity at 16 weeks gestation, metabolic hormones such as insulin; gastric inhibitory polypeptide; and adipokine were correlated with the relative abundance of maternal *Collinsella; Coprococcus* and *Ruminococcaceae;* and *Ruminococcaceae* and *Lachnospiraceae*, respectively, reinforcing the relationship betwee*n* gut microbiome composition and the metabolic hormonal environment (Gomez-Arango et al., 2016).

Clinical studies that examine microbiome composition in both pregnant individuals and their offspring are few, and often heavily depend on self-reported food-questionnaires that may not comprehensively capture dietary intake at all phases of pregnancy. These include, for instance, a cohort of 86 mother-neonate pairs, in which maternal microbiota composition shortly before birth distinctly grouped into two clusters—one characterized by *Prevotella* (Cluster I) and the other by the *Ruminococcus* genus (Cluster II)—based on maternal diet (García-mantrana et al., 2020). Higher intakes of total dietary fiber, omega-3 fatty acids, and polyphenols were more prevalent in Cluster II compared to Cluster I (García-mantrana et al., 2020). Maternal microbial clusters were associated with neonatal microbiota and infant growth in a mode-of-delivery-dependent manner, with infants of Cluster I mothers delivered via C-section showing higher BMI and weight-for-length (WFL) z-scores at 1 and 18 months (García-mantrana et al., 2020). Similarly, in a Norwegian birth cohort study of 169 mothers and their 181 children, significant changes in maternal gut microbial composition were noted at time of delivery in mothers with pre-pregnancy overweight/obesity (BMI ≥ 25) (Stanislawski et al., 2017). Pre-pregnancy BMI≥ 25 or excessive gestational weight gain was associated with taxonomical differences in the family *Christensenellaceae* and the genera *Lachnospira, Parabacteroides, Bifidobacterium*, and *Blautia* in mothers (Stanislawski et al., 2017). While these characteristics were not associated with overall differences in offspring gut microbiota over the first two years of life, the presence of specific operational taxonomic units in the maternal gut microbiota significantly increased the odds of their presence in the infant gut at age 4–10 days for many taxa, including lean-associated taxa (Stanislawski et al., 2017). Other human studies describing microbial compositional shifts in offspring born to mothers with HFD have consistently shown that offspring gut microbiome composition varies with maternal HFD/obesity (Collado et al., 2010; Galley et al., 2014; Chu et al., 2016; Garcia-Mantrana and Collado, 2016), with still others placing a role for fruit and dairy consumption in altering clustering of infants to specific microbial compositions (Lundgren et al., 2018). However, some dietary exposures do not appear to have dramatic effects on the offspring microbiome. For instance, in the ALADDDIN birth cohort of 128 mother-infant pairs, significant differences in microbial composition (namely increased *Bifidobacterium* and lower abundance of *Bacteroides* and *Veillonella*) were found in offspring of mothers with an anthroposophic lifestyle, but only after 6 months of age (Hesla et al., 2014). Importantly, birth mode emerged as the primary determinant of infant and maternal microbiota, rather than the anthroposophic lifestyle (Hesla et al., 2014). It is worth noting that these data were generated using pyrosequencing, a method with lower sensitivity and resolution compared to modern sequencing techniques. The application of newer sequencing technologies, such as high throughput 16S rRNA gene sequencing or metagenomics, could potentially reveal additional microbial differences that may not have been detectable with earlier methodologies, as demonstrated in other studies of farming and anthroposophic lifestyles.

In animal models, gestational HFD similarly elevates Bacillota and decreases Bacteroidota, in mothers, with some studies reporting further increases in *Akkermansia* and *Clostridium* and decreases in *Lachnospira* and *Ruminococcus* (Ley et al., 2005; Mann et al., 2018; Gohir et al., 2019; Takeuchi et al., 2022; Urbonaite et al., 2022). Preclinical studies utilizing a variety of animal models, including non-human primates (Ma et al., 2014), mice (Bruce-Keller et al., 2017; Babu et al., 2018; Zhou et al., 2020), and rats (Srinivasan et al., 2006) have further shown microbial dysbiosis and metabolic disorders in offspring at weaning provoked by maternal HFD. This was associated with hyperglycemia, glucose intolerance, and insulin intolerance in the offspring at weaning in some reports (Sun et al., 2012; Guo et al., 2018; Zhang et al., 2019; Zheng et al., 2022), with one study showing effects on offspring metabolism persisting at 52 weeks of age (Stanford et al., 2017). In some studies, exercise (Srinivasan et al., 2006; Stanford et al., 2017; Zhou et al., 2020) or prebiotic/probiotic administration (Hallam et al., 2014; Paul et al., 2016; Cheng et al., 2018; Zhang et al., 2019) in dams had some improvement in microbiome composition and metabolic profile, though this was not consistent across studies. Additionally, studies have diverged on the impact of maternal HFD on offspring metabolism post-weaning when offspring are delivered a normal diet. While some research has indicated that offspring transition to a normal diet can offset gestational maternal HFD (Zheng et al., 2016; Akhaphong et al., 2022; Zheng et al., 2022), others have found that regardless of offspring post-weaning diet, metabolic deficits or alterations in the microbiome still persist (Myles et al., 2013; Ma et al., 2014; Bhagavata Srinivasan et al., 2018; Guo et al., 2018; Xie et al., 2018; Zhou et al., 2020). For instance, Ma et al., demonstrated that *Campylobacter*, a commensal in Japanese macaques, was persistently diminished in the HFD offspring regardless of post-weaning diet (Ma et al., 2014). Additionally, others have shown microbial alterations still persist even if metabolic profile is not significantly different to controls, which might have a role in the promotion of susceptibility to obesity and diabetes later in life (Zheng et al., 2022).

Collectively, these studies show that the extent to which maternal HFD may induce measurable changes to offspring gut microbiota may differ based on etiology of obesity during pregnancy (Galley et al., 2014), pregnancy trimester (Hesla et al., 2014; Stanislawski et al., 2017; García-mantrana et al., 2020), and method of delivery (Hesla et al., 2014; Mueller et al., 2016; García-mantrana et al., 2020). Current recommendations from the American College of Obstetrics and Gynecology (ACOG) encourage maintaining a BMI within the normal range, and conducting dietary screening to ensure pregnant individuals obtain the recommended daily allowances for diet and vitamin supplements (Mirpuri, 2021). However, the optimal composition of macronutrients during pregnancy remains unclear, including how specific macronutrient profiles may influence maternal health, fetal development, and metabolic outcomes in offspring. Sex-specific effects of dietary interventions further remain to be examined. For instance, maternal vitamin D was found to program colonic *Bacteroides* in male offspring only, which negatively correlated with systemic inflammation and positively with bone strength and structure (Villa et al., 2018). As maternal diet is a modifiable risk-factor during pregnancy, elucidating the effects of maternal diet and obesity during pregnancy on offspring gut microbiome and metabolic profile, in particular, may provide a window of opportunity for therapeutic intervention.

### Other maternal conditions

Several maternal conditions have been identified that influence the composition of microbiota transferred to offspring at birth. Among these, maternal stress, intestinal disorders, and gestational diabetes mellitus have been well-investigated.

Stress is well-known to influence gut microbiota composition through changes in gut physiology, including reducing the production of gastric acid, preventing bile release from the gallbladder to the small intestine, reducing small intestinal motility, and altering levels of secretory IgA (Yeramilli et al., 2023). Accordingly, stress during pregnancy may result in gut dysbiosis in offspring in both preclinical (Bailey and Coe, 1999; Jašarević et al., 2015; Jašarević et al., 2018; Gur et al., 2019; Brawner et al., 2020) and clinical studies (Zijlmans et al., 2015). This is often associated with alterations in the metabolite profile in the periphery and the brain (Jašarević et al., 2015; Jašarević et al., 2017; Jašarević et al., 2018). In some studies, alterations in both the maternal gut and vaginal microbiota in pregnant mice exposed to stress were associated with parallel reductions of *Lactobacillus* abundance in neonates (Jašarević et al., 2015; Jašarević et al., 2017). Transplantation of vaginal microbiota from stress-exposed females into naïve offspring produced a similar phenotype as that observed in prenatally stress-exposed offspring, including altered microbiota composition and body weight, as well as increased corticosterone release in response to an acute stressor (Jašarević et al., 2018). However, prenatal stress effects on body weight and corticosterone response to acute stress were not rescued by transplantation of vaginal samples from control dams into prenatal-stress exposed offspring; this was attributed to transcriptomic reprogramming of the fetal intestine prior to birth (Jašarević et al., 2018). In particular, male offspring may be more vulnerable to the effects of prenatal stress. Following prenatal stress, male offspring display alterations in gene expression in the paraventricular nucleus of the hypothalamus (Jašarević et al., 2018); exhibit increased corticosterone release in response to stressful stimuli compared to adult males born from non-stressed women (Gur et al., 2019); and show decreased placental expression of X-linked-O-linked Nacetylglucosamine transferase, a nutrient sensing enzyme in the placenta (Howerton et al., 2013). Mechanisms by which prenatal stress can influence offspring health are multifaceted and may overlap with other maternal exposures that result in gestational dysbiosis. Fetal exposure to excessive glucocorticoids can modulate fetal hypothalamus-pituitary-adrenal (HPA) axis feedback by increasing placental corticotropin-releasing factor production and signaling (Mastorakos and Ilias, 2009). Additionally, maternal stress can promote an inflammatory state in the placenta leading to desensitization of the HPA axis, particularly in male offspring (Bronson and Bale, 2014). Stress-induced glucocorticoid activity can also modulate the maternal and offspring immune system, resulting in changes in the migration, differentiation, and proliferation of immune cells (Herrera et al., 1988; Garcia-flores et al., 2020; Yeramilli et al., 2023). Furthermore, prenatal stress may also alter placental function and signaling by inducing epigenetic changes. Currently reported are increased promoter methylation of 11β-hydroxysteroid dehydrogenase type 2 (11βHSD2) and the expression of DNA methyltransferase 3A in the placenta (Benediktsson et al., 1997; Jensen Peña et al., 2012), which is associated with decreased transformation of maternal cortisol into inactive cortisone. This has been recapitulated in human placentas obtained from women with anxiety/depression, in which placental epigenetic modifications positively corelated with impairments in infant neurobehavior (Conradt et al., 2013; Appleton et al., 2015).

Additionally, conditions like inflammatory bowel disease (IBD), irritable bowel syndrome, and celiac disease—all of which result in chronic gut inflammation—disrupt the balance of microbial communities. In fact, Torres et al., reported that maternal IBD status was the main predictor of infant gut microbiota diversity at 7, 14, 30, 60, and 90 days of life (Torres et al., 2020). Pregnant women with IBD exhibited lower α-diversity during the first and second trimester of pregnancy (Torres et al., 2020). Additionally, they had altered β-diversity which was driven by a depletion in the relative abundance of Bacteroidota and an increase in the relative abundance of Pseudomonadota (Torres et al., 2020), which has been associated with intestinal inflammation and IBD in multiple reports (Morgan et al., 2012). In parallel, offspring of women with IBD were enriched in *Gamma*Pseudomonadota and depleted in *Bifidobacteria*, consistent with patterns observed in adult and childhood-onset IBD patients (Gevers et al., 2014; Kostic et al., 2014). Notably, germ-free mice inoculated with either third trimester stool from IBD women or 90-day infant stool had significant reduction in microbial diversity, IgA class-switched memory B cells, and regulatory T cells in the colon (Torres et al., 2020). Consistently, patients with IBD have impaired IgA production (Uzzan and Colombel, 2016). Notably, IgA is the predominant antibody at mucosal surfaces, and is key in downregulating pro-inflammatory epitopes on commensal bacteria, upregulating growth of commensal microbiota, directing luminal bacteria to M cells, and promoting the maturation of dendritic cells and the production of IL-10 (Corthésy, 2013).

Last, gestational diabetes mellitus (GDM) exerts a unique influence on the maternal microbiome during pregnancy, which further influences the infant gut. GDM is diagnosed in weeks 24 to 28 of pregnancy by oral glucose tolerance test and is marked by insulin resistance first occurring during pregnancy (McIntyre et al., 2019). Upwards of 10% of pregnancies in the US are complicated by GDM, and risk factors include obesity, pre-diabetes, a previous history of gestational diabetes, polycystic ovary syndrome, and a family history of diabetes (McIntyre et al., 2019). Studies have shown that GDM is associated with abnormalities in the maternal gut microbiota (Crusell et al., 2018; Ferrocino et al., 2018; Cortez et al., 2019; Zheng et al., 2020) and both the maternal and neonatal gut microbiota (Wang et al., 2018; Qin et al., 2022). Significant changes in the phyla Bacillota, Bacteroidota, Actinomycetota, Pseudomonadota*, Verrucomicrobiota*, and *Fusobacteriota* have been recorded in the gut microbiome of women with GDM, though their relative abundances vary across studies (Farhat et al., 2022). Similar variation was found in the gut microbiome of offspring born to women with GDM (Farhat et al., 2022). Still, these alterations in the offspring gut microbiome were observed to relatively mirror those of their mothers with GDM (Farhat et al., 2022). Alternatively, in mice, FMT of GDM-feces into germ-free mice lowered the relative abundance of *Akkermansia, Faecalibacterium* and SCFA; and elevated blood glucose, hepatic fat deposition, and the inflammatory profile (TNF-α, CXCL-15, and IL-6) in dams (Qin et al., 2022). In parallel, offspring exhibited gut dysbiosis and had higher body weight and blood glucose levels relative to control offspring (Qin et al., 2022). In other studies, vertical transmission of a GDM-microbiome has also been associated with an increased risk of diabetes in the offspring of GDM women (Farahavar et al., 2019). Again, these data corroborate the observation that the inheritance of a dysbiotic gut microbiome may increase the risk of future disease in offspring.

## Current theories for how the maternal GI tract influences neonatal gut microbial seeding

**Figure 4:**
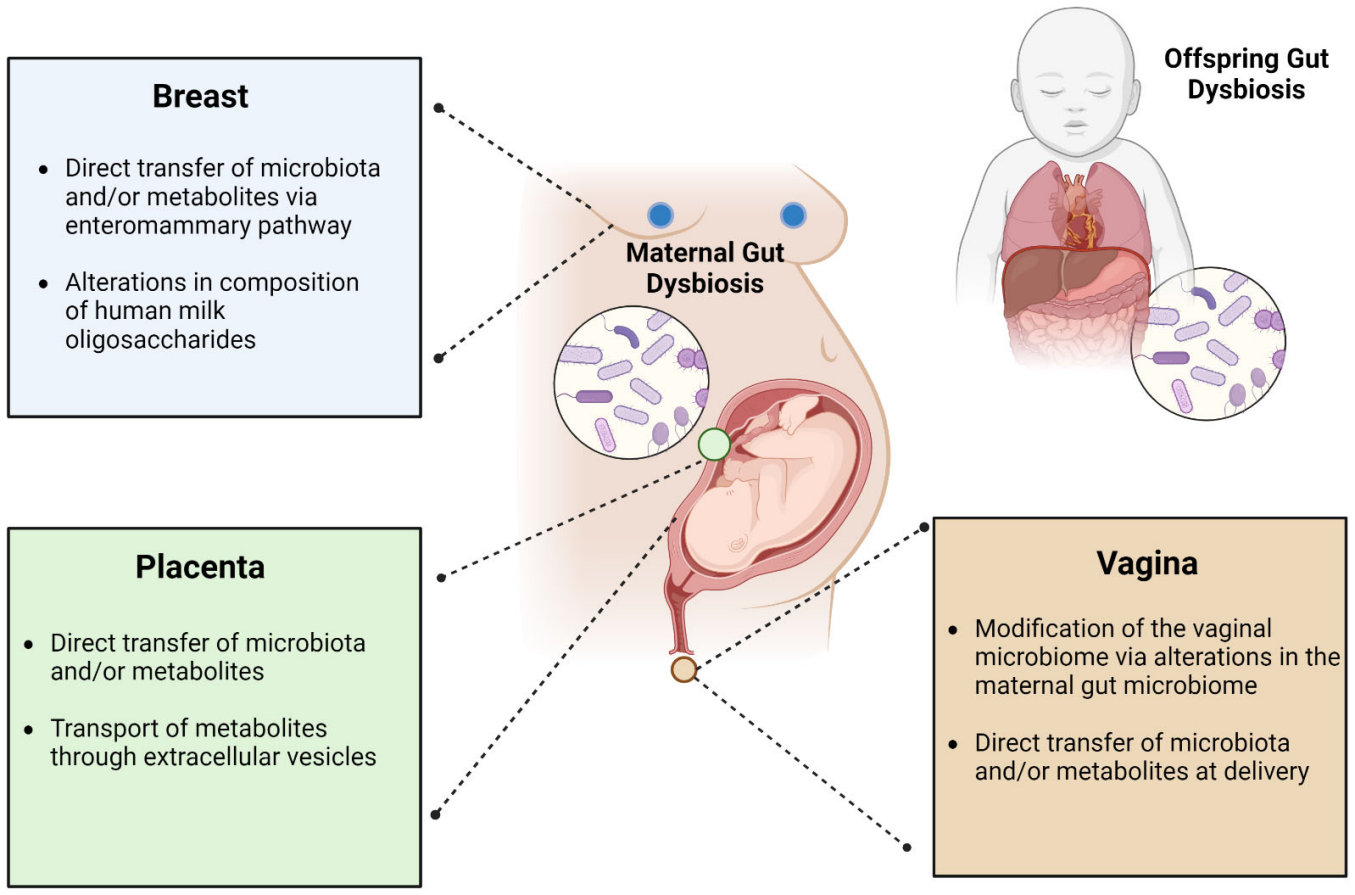
Leading theories describing the influence of maternal gut dysbiosis during pregnancy on the gut microbiome of offspring. The maternal gut microbiome is hypothesized to impact the offspring gut microbiome through its interactions with the breast, placenta, and/or vagina. Its influence via the breast may occur through direct transfer of microbiota and/or metabolites through the enteromammary pathway or gut-microbiome mediated alterations in the composition of human milk oligosaccharides. From the placenta, this may occur through direct transfer of microbiota and/or metabolites or may be mediated through extracellular vesicles. From the vagina, this is thought to occur through gut microbial modification of the vaginal microbiome or through direct transfer of microbiota and/or metabolites in the peripartum period.

While casual pathways for neonatal gut microbiome seeding have not been elucidated, some studies have posited that seeding may be influenced prenatally though placental microbiota/metabolite transmission and/or postnatally via interactions with the mother’s vagina/feces, the entero-mammary pathway, or breast milk oligosaccharides (**Figure 4**).

### *In utero* contributions: transfer of bacteria or bacterial metabolites

While the womb was long thought to be sterile, this view has been challenged over the past decade, with claims that microbiota colonize the placenta (Zakis et al., 2022). Initial evidence came from DNA-based, culture independent studies detecting microbial DNA in placental tissue (Aagaard et al., 2014; Collado et al., 2016), amniotic fluid (Collado et al., 2016; Liu et al., 2019; He et al., 2020), and meconium (Jiménez et al., 2008; Hu et al., 2013; Ardissone et al., 2014; Hansen et al., 2015; Chu et al., 2017; Liu et al., 2019; Stinson et al., 2019), which blossomed the *in utero* colonization hypothesis. For instance, analysis of samples from full-term cesarean births revealed microbial features dominated by Pseudomonadota, shared between placenta, amniotic fluid, and meconium, suggesting microbial transfer at the maternal-fetal interface (Collado et al., 2016). Additionally, *ex vivo* studies showed that labelled *Enterococcus faecium* strains (isolated from human breast milk) could be PCR-detected in the meconium of cesarean-section delivered offspring (Jiménez et al., 2008). Similarly, a preclinical study showed that orally-administered foreign bacteriophage DNA could be detected in fetuses and newborn animals (Hohlweg and Doerfler, 2001). Together, these studies presented a major break from the existing dogma that bacterial colonization is observed only in the context of preterm birth or with infection of fetal membranes/amniotic fluid, rather than at these sites in healthy pregnancies (Mshvildadze et al., 2010; Ma et al., 2014; Prince et al., 2016). However, recent studies have reported conflicting findings, with microbial DNA placental and meconium samples (low biomass sites) attributed to contamination (de Goffau et al., 2019; Kennedy et al., 2021; Dos Santos et al., 2021). Critics note the inability to consistently culture microbes from healthy placentas, the unresolved mechanism for fetal microbiome control with immature immune systems, and the development of germ-free offspring via cesarean delivery, which seems incompatible with prenatal colonization (Blaser et al., 2021). Lastly, others have noted that findings of bacterial DNA are distinct from actual bacterial colonization, defined as a stable community over time that is metabolically active and reproducing *in situ*. Nevertheless, other studies accounting for contamination controls (e.g., extraction buffers, procedural swabs, hospital room air swabs, blank cotton swabs, or fetal kidney controls) have been able to detect limited bacterial DNA in the human fetal intestine (Rackaityte et al., 2020). With these mixed data, the bar for proving colonization is high.

As an alternative, transplacental transport of microbiota-derived metabolites (SCFAs) or compounds has gained wider acceptance. While the intestinal epithelial barrier normally prevents microbes and other contents from entering the bloodstream (Amir et al., 2020), animal models have shown increased permeability of this barrier during pregnancy (Gohir et al., 2019; Wallace et al., 2019). This could be experimentally exacerbated with animals fed HFDs during pregnancy (Gohir et al., 2019; Wallace et al., 2019), resulting in increased circulating levels of maternal LPS and tumor necrosis factor, altered metabolome in the placentas of these dams, and increased activation of NF-kB in fetuses born to these dams (Gohir et al., 2019). Interestingly, the increased maternal gut permeability induced by HFDs could be reversed by prebiotic treatment, which led to an increased abundance of *Bifidobacterium* species in the gut, though this was not in the context of pregnancy (Cani et al., 2009). Maternal HFD in another study was shown to lower the abundance of *Lactobacillus reuteri* in the maternal gut microbiota, which reduced oxytocin levels in the hypothalamus of offspring and negatively affected social behavior, presumably through vagal nerve communication (Buffington et al., 2016); this was transferable to germ-free mice and prevented postnatally by co-housing with offspring of mothers on a regular diet (Buffington et al., 2016). Cross-placental transfer of microbial metabolites was again shown in a study that analyzed the placenta, fetal intestine, and brain samples from germ-free and specific pathogen free murine dams using a broad non-targeted metabolomics approach, which revealed 3680 differentially molecular features (Pessa-Morikawa et al., 2022). Of these, germ-free fetal organs exhibited significantly lower 5−aminovaleric acid betaine (5-AVAB), trimethylamine N−oxide (TMAO), catechol−O−sulphate, hippuric acid, and pipecolic acid (Pessa-Morikawa et al., 2022). Consistently, others have shown that select microbiota-dependent metabolites such as TMAO, 5-AVA, 5-AVAB, imidazolepropionic acid, and hippuric acid could promote axonogenesis and abrogate deficiencies in fetal thalamocortical axons both *in vitro* and *in vivo*, in the absence of live bacteria (Vuong et al., 2020). Cumulatively, these studies suggest that the maternal microbiome can influence the fetal metabolome and fetal development independent of microbial transfer.

Still, there are other mechanisms at play prenatally that are being evaluated. One such mechanism has stemmed from the observation that during term labor, Lactobacillus and bacterial vaginosis-associated bacteria can ascend from the vagina to the chorioamnion even in the absence of chorioamnionitis (Lannon et al., 2019). Additionally, recent studies propose maternal microbiota-derived extracellular vesicles (EVs) as a means of communication between the maternal microbiome and the fetus. Bacterial EVs have the capacity to cross the gut epithelial barrier into systemic circulation and reach distant sites, thereby representing a long-distance microbiota–host communication system (Jang et al., 2015; Park et al., 2017; Jones et al., 2020; Kim and Yi, 2020). In a cohort of 28 pregnant women undergoing elective cesarean section delivery after a term pregnancy, microbiota-derived EVs were found in the amniotic fluid (Kaisanlahti et al., 2023). These EVs exhibited similarities to EVs found in the maternal fecal samples in terms of their protein cargo and bacterial composition, suggesting a common source (Kaisanlahti et al., 2023). In mouse models, fluorescently labeled EVs derived from the fecal samples of pregnant women injected into the tail vein of pregnant mice were detected in fetuses and distant maternal organs, demonstrating their ability to cross the placental barrier (Kaisanlahti et al., 2023). Others have further shown via electron microscopy that the microbiome of the first-pass meconium samples contains bacterial EVs (Turunen et al., 2023). These EVs had bacterial RNA from phyla Bacillota (62%), Actinobacteriota (18%), Pseudomonadota (10%), and Bacteroidota (7.3%) (Turunen et al., 2023). However, their origin remains unclear (Turunen et al., 2023), with the possibility of perinatal colonization or intrauterine colonization via bacterial EVs derived from the mother more likely based on the absence of placental or amniotic fluid microbiota in their earlier reports (Turunen et al., 2021). Further research is needed to clarify the mechanisms and implications of these maternal influences on fetal development.

### Contributions from the vaginal microbiome

The vaginal microbiota is undoubtedly an important contributor to the gut microbiome of vaginally-born infants. For instance, vaginal microbiomes are primarily dominated by *Lactobacillus*, which represent a major genus in the gut of vaginally-born (compared to C-section delivered) infants (Mueller et al., 2015). Furthermore, increased abundance of facultative anaerobic species such as *Escherichia coli*, *Staphylococcus*, and *Streptococcus* that colonize the infant gut and produce anaerobic environments have commonly been associated with vaginal birth, which further support the growth of strict anaerobes such as *Bacteroides* and *Bifidobacterium* spp (Mueller et al., 2015). Conversely, C-section babies have been described to lack vaginal microbes (e.g., *Lactobacillus, Prevotella, Sneathia* spp.) at birth, and instead harbor an abundance of skin bacteria (e.g., *Streptococcus, Staphylococcus*, *Corynebacterium, Propionibacterium* spp.), intestinal *Clostridium difficile*, and opportunistic pathogens found in hospital environments (e.g., *Enterococcus*, *Enterobacter*, and *Klebsiella* species) (Mueller et al., 2015; Hill et al., 2017; Tanaka and Nakayama, 2017; Dalby and Hall, 2020; Wernroth et al., 2022). C-section offspring are also reported to experience delays in postnatal colonization of intestinal *Bacteroides* and *Bifidobacterium* spp (Mueller et al., 2015; Dalby and Hall, 2020). Interestingly, immediate postnatal interventions such as vaginal microbiota transfer (VMT) following C-section delivery have been shown to accelerate gut microbiota maturation and improve developmental outcomes in offspring, suggesting vaginal microbiota regulation of certain gut metabolites and metabolic pathways (Zhou et al., 2023).

Despite the significant compositional differences identified across a large number of studies, there has been inconsistent evidence surrounding species/strains involved as well as whether this initial dysbiosis may persist (Dos Santos et al., 2021). Additionally, certain observations also challenge the conventional understanding of mode of delivery as a major contributor to offspring gut microbial seeding. Principal among these is the substantial variability in vaginal microbiome composition that exists amongst women and the failure of studies to thus demonstrate associations between vaginal microbiome and infant gut microbial profiles if the vaginal microbiome is indeed seeding the infant gut microbiome (Dos Santos et al., 2023a). This variability in associations may reflect that both the vaginal and gut microbiomes contribute to initial microbial seeding and succession in infants, rather than one being the sole driver. The interplay between these maternal microbial niches could explain the lack of strong associations observed between the vaginal microbiome and infant gut microbiome in some studies. Additionally, others have pointed out that existing studies do not differentiate between elective and emergency C-section, even though the latter has prolonged exposure to vaginal microbiota due to prolonged rupture of fetal membranes, which may confound analysis (Dos Santos et al., 2023a).

In some reports, it appears that the maternal gut microbiota, rather than the maternal vaginal microbiota, may be more predictive of the matured offspring gut microbiome (Torres et al., 2020; Fricke and Ravel, 2022; Dos Santos et al., 2023a). Maternal vaginal strains are reported to contribute only a small and transient fraction of the neonatal intestinal microbiota, with maternal intestinal microbiota contributing a significantly larger proportion (Ferretti et al., 2018; Stinson et al., 2018; Mitchell et al., 2020; Podlesny and Fricke, 2021). For instance, maternal vaginal microbiome composition was not a major predictor of the composition of the infant stool microbiome at 10 days or 3 months of life, regardless of delivery mode (Dos Santos et al., 2023a; Dos Santos et al., 2023b). This is further recapitulated by the limited success of oral administration of maternal vaginal microbes at birth to restore gut microbiome development in infants born by cesarean section (Wilson et al., 2021). Additionally, others have found that differences in microbial composition between vaginally or C-section delivered offspring often disappear with the maturation of the infant microbiota towards an adult state between 2 to 5 years of age (Chu et al., 2017; Stewart et al., 2018; Podlesny and Fricke, 2021; Roswall et al., 2021). On the other hand, detectable microbiota signatures in 5- to 7-year-old children born by C-section have still been reported (Salminen et al., 2004; Roswall et al., 2021). It is possible that some vaginal microbes may be transferred vertically to the neonatal gut to affect microbial seeding. However, existing studies supporting these claims often are lacking in direct evidence of maternal vaginal organisms or in demonstration of the absence of vertical transfer in C-section delivered infants (Dos Santos et al., 2023a; Dos Santos et al., 2023b). Thus equally plausible is the notion that maternal microbial transmission may arise from other maternal body sites, such as the gut and breast milk, that also harbor microbiota also found in the vagina such as *Bifidobacterium* or *Enterococcus* (Dos Santos et al., 2023a; Dos Santos et al., 2023b).

### Gut-breast milk-bacterial axis: entero-mammary pathway

There is evidence of translocation of small amounts of bacteria present in the maternal digestive tract to the mammary gland during pregnancy and lactation (Asnicar et al., 2017; Ferretti et al., 2018; Yassour et al., 2018; Zhong et al., 2022). Previous studies have demonstrated the presence of two lactic acid bacteria strains (*Lactococcus lactis MG1614 and Lactobacillus salivarius PS2*), transformed with a plasmid containing the lux genes, in milk and mammary gland biopsies after their oral administration to pregnant mice (de Andres et al., 2017). Similarly, *L. salivarius PS2* was detected in the milk of women after delivery after its oral administration in women during pregnancy (Fernández et al., 2016). More recently, Zhong et al., recruited 11 healthy mother-infant pairs, in which pregnant women ingested a biomarker strain, *Bifidobacterium animalis subsp. lactis Probio−M8* (M8), daily to assess translocation of maternally ingested bacteria to the infant gut via lactation using both traditional culture methods and high-throughput sequencing (Zhong et al., 2022). The M8 strain was directly recovered in most breast milk and some infant fecal samples; of the 11 mother-infant pairs, 5 infant fecal samples and 1 breast milk did not return any target bacterial clones (Zhong et al., 2022). Interestingly, intra-strain diversity and metabolic phenotype analyses further showed that in mother-infant pairs with recovered M8 homologous isolates, these bacteria could adapt to new environmental niches via genomic polymorphism, especially in the sugar transporter glcU gene (Zhong et al., 2022). These strains further exhibited altered carbohydrate utilization compared with non-mutant isolates, suggesting that M8 underwent adaptive evolution for better survival in simple sugar−deprived lower-gut environments (Zhong et al., 2022). Of note, in this study, a high probiotic dose was used to enhance detection of the target strain, as a standard daily dose returned a non-statistically significant number of recovered M8 homologous strains (Zhong et al., 2022).

The scientific basis for gut bacterial translocation to the mammary glands has not been clarified; it is thought to occur through gut bacterial uptake by intestinal dendritic cells followed by migration to mesenteric lymph nodes (MLN), allowing these bacteria to enter the urogenital system, respiratory mucosa, saliva, and lactating breasts (Macpherson and Uhr, 2004). Commensal bacteria are rapidly killed by macrophages, but it was found that intestinal dendritic cells can retain small numbers of live commensals for several days restricted to the mucosal immune compartment by mesenteric lymph nodes (Macpherson and Uhr, 2004; Perez et al., 2007). It has been suggested that this may be a physiological process during pregnancy. Bacterial translocation to extraintestinal tissues was assessed in non-pregnant, pregnant, and lactating mice, and whereas 10% of non-pregnant animals had positive MLN cultures, 70% of pregnant animals had bacteria in their MLNs. Within 24 hours after delivery, 80% of mice had viable bacteria in their mammary tissue; this percentage decreased each day following delivery, though it still remained significantly higher than in that of non-pregnant control mice four days post-partum (Perez et al., 2007). Additionally, Peyer’s patches (PPs) of pregnant and lactating mice were found to be macroscopically larger than those of non-pregnant animals and exhibited more dilated draining lymphatic vessels, containing mononuclear cells (Perez et al., 2007).

Other studies also posit the potential role of secretory immunoglobulin A (sIgA) in the entero-mammary pathway, whose translocation to milk has recently been shown to be influenced by microbiota residing in PPs. sIgA is the dominant immunoglobulin residing in the mucosal surface of the intestinal tract and is able to recognize and coat bacteria as well as LPS, capsular polysaccharides, and flagellin on the bacterial surface (Ding et al., 2021). While research in the field of sIgA-coated bacterial profiles in human milk is still at its infancy, sIgA-coated *Bifidobacterium* and *Lactobacillus* co-occurrence in human milk and infant intestine have been observed (Ding et al., 2021), suggesting sIgA-coating as another manner for bacterial transmission through entero-mammary pathways. Recently, one study uncovered that the commensal gut bacteria *Bacteroides acidifaciens* and *Prevotella buccalis* can induce PP-dependent production of sIgA into breast milk, garnering support for a microbiota-PP-mammary gland pathway (Usami et al., 2021). Lactating PP-intact and PP-null mice were orally dosed with either *B. acidifaciens* or *P. buccalis*, and both the number of IgA plasma cells in the mammary glands and the level of maternal IgA in milk were significantly increased in PP-intact mice dosed with either *B. acidifaciens* or *P. buccalis*, but not in PP-null mice even when they were orally dosed with either *B. acidifaciens* or *P. buccalis*. This highlights the role of PPs and the influence of microbiota (Usami et al., 2021). Collectively, these studies have demonstrated that bacteria present in human milk may originate in the maternal intestine and enter the mammary gland via entero-mammary pathways.

## Probiotics during pregnancy

With the maternal gut microbiome being increasingly recognized as a major contributor to offspring health, current strategies to enhance neonatal outcomes are focused on maintaining or restoring microbial homeostasis during pregnancy via probiotics, prebiotics, or synbiotics. Among these, probiotics—defined as live microorganisms that, when administered in adequate amounts, confer health benefits on the host—are the most commonly employed to improve both maternal and neonatal health. Although concerns exist regarding host-microbe interactions and side effect profiles, the few double-blinded, randomized, placebo-controlled trials have shown that probiotic administration during pregnancy and the neonatal period is generally safe (Allen et al., 2010; Luoto et al., 2010), with more adverse effects reported in the immunocompromised, critically ill, and those with pre-existing GI pathology (Baldassarre et al., 2018). Despite the absence of an FDA-approved pharmaceutical-grade probiotic, commercial use during pregnancy and supplementation of infant formula with probiotics continue to rise (Poindexter and COMMITTEE ON FETUS AND NEWBORN, 2021); however, the efficacy of these products on neonatal health outcomes is highly contentious (Braegger et al., 2011).

Multiple studies have investigated the effect of probiotics taken by pregnant or breast feeding women and/or their infants on the prevalence of infection, preterm delivery, obesity, necrotizing enterocolitis, and allergic diseases (Baldassarre et al., 2018). While some meta-analyses have found a protective role for probiotic supplementation, others have found no discernable differences in neonatal outcomes (Cuinat et al., 2022). For instance, though some human studies evaluating the effect of maternal probiotic administration on infant outcomes have noted some improvement in atopic disease (Avershina et al., 2017; Peldan et al., 2017; Rø et al., 2017; Wickens et al., 2018a), colic frequency (Pourmirzaiee et al., 2020), immune maturation (Forsberg et al., 2020), and macrosomia (Sahhaf Ebrahimi et al., 2019) in neonates, others have found no such effect (Dewanto et al., 2017; Davies et al., 2018; Kallio et al., 2018; Wickens et al., 2018b). Importantly, most human studies have not assessed the influence of maternally administered probiotics on infant gut microbiota composition or rather have focused on general probiotic administration rather than targeting underlying dysbiosis directly, which may limit their capacity to induce meaningful changes in microbiome composition and function. This could explain the lack of substantial, consistent effects on neonatal health outcomes. On the other hand, those that have evaluated microbiota composition report only transient increases in the abundance of maternally administered probiotics in offspring. For example, Dotterud et al., reported that administration of a mixture of *Lacticaseibacillus rhamnosus GG, Bifidobacterium animalis subsp. lactis Bb-12*, and *Lactobacillus acidophilus La-5* in pregnant women from 36 weeks of gestation until 3 months after birth did significantly increase *Lactobacillus rhamnosus GG* in offspring assessed at 3 months of age (Dotterud et al., 2015). Similarly, Korpela et al., reported that administration of a mixture of *Bifidobacterium breve Bb99, Propionibacterium freudenreichii subsp. shermanii JS, Lactobacillus rhamnosus Lc705*, and *Lactobacillus rhamnosus GG* in both pregnant women and their infants increased *Bifidobacterium breve* and *Lacticaseibacillus rhamnosus* in 3-month-old antibiotic-treated or cesarean-born infants (Korpela et al., 2018). This effect was dependent on the infant’s diet, with breastfed infants showing the expected increase in *Bifidobacteria* and reduction in Pseudomonadota and *Clostridia* (Korpela et al., 2018).

Preclinical studies evaluating probiotic administration on infant outcomes have largely been able to inform of changes to microbiome composition and diversity in offspring (Cuinat et al., 2022). These studies have found mixed results, with some finding increased (Xiao et al., 2020), decreased (Zhao et al., 2022), or no changes (Hsu et al., 2019; Azagra-Boronat et al., 2020; Wang K. et al., 2021; Han et al., 2022; Qi et al., 2022; Zhou et al., 2022) in α-diversity. Similarly, while some studies have reported significant differences (Wang K. et al., 2021; Han et al., 2022; Krishna et al., 2022; Qi et al., 2022; Zhou et al., 2022) in β-diversity of infant gut microbial samples following maternally administered probiotics during pregnancy, others have not detected any discernable differences (Hsu et al., 2019; Azagra-Boronat et al., 2020). Whether changes to microbial diversity and composition in neonates following maternal probiotic administration are necessary to improve neonatal outcomes is unknown. While fecal samples from 3-week-old offspring whose mothers had received *Lacticaseibacillus casei* during pregnancy did not show any differences in α- or β-diversity compared to controls, remarkably the probiotic intervention was able to prevent HFD-induced decrease in *Alkaliphilus* and *Akkermansia* abundances and several *Lactobacillus* species (Hsu et al., 2019). Prevention of high-fat-diet-induced elevation of BP was associated with a reduction in plasma trimethylamine (TMA) and the TMAO to TMA ratio, as well as a reduction in fecal propionate and acetate levels (Hsu et al., 2019). This emphasizes that probiotics may also modulate microbial function through alterations to the metabolome, which can be explored using metagenomic approaches.

Consensus on probiotic efficacy in these studies is often complicated by heterogeneity in probiotic species and dose; single versus multiple strains included in the probiotic; administration of probiotic; duration and timing of intervention; and study design, limiting comparability of the results. Wickens et al., further underscored the differential efficacy of probiotic species: *Lactobacillus rhamnosus HN001* administered in the peripartum period and to infants daily until age 2 was found to reduce the prevalence of asthma, whereas in the same trial the probiotic *Bifidobacterium animalis subs. lactis HN019* did not provide significant protection against eczema or atopic sensitization (Wickens et al., 2018b). Furthermore, the differences between maternal probiotic administration alone and combined maternal and infant supplementation make it difficult to determine whether the benefits of probiotics arise from the maternal transfer of protective factors, direct supplementation to the infant through breast milk, or a combination of both (Swartwout and Luo, 2018). Timing of probiotic administration is also critical, as interventions may need to begin during pregnancy to influence fetal immune programming effectively. These considerations underscore the importance of further research on the optimal timing and method of probiotic administration, as well as the need to investigate how maternal supplementation could influence the infant microbiome and immune development *in utero*.

Difficulties establishing the benefits of probiotics in preventing pediatric disease also arise from problems achieving stable engraftment; variability in duration of probiotics between studies also contributes to conflicting results between studies. Most studies that attempt to alter intestinal dysbiosis via probiotic administration have only been able to achieve transient engraftment, with bacterial communities returning to baseline shortly after discontinuation of the probiotic (Tamburini et al., 2016). For instance, at 1 and 2 years of age, Dotterud et al., reported no significant differences in the abundance of maternally administered probiotics between the probiotic intervention group compared to placebos (Dotterud et al., 2015). Additionally, α- and β-diversity were not significantly different between intervention and control groups at 3 months or 2 years of age, though improvements in atopic disease were noted at earlier timepoints in the intervention group (Dotterud et al., 2015). However, other studies have shown durable changes in the human gut microbiome well after the supplementation period (Frese et al., 2017). Frese et al., conducted a pivotal study that assessed the direct probiotic supplementation to infants, demonstrating that breastfed infants were stably colonized by high levels of *Bifidobacterium infantis* EVC001, which persisted for up to a year following the supplementation period. This effect demonstrates the potential value of pairing a probiotic organism with a specific substrate; in this case, *B. infantis* EVC001 was paired with human milk glycans in breast milk as the source of probiotic therapy. Key in this study was a focus on probiotic supplementation directly to infants, which differs substantially from the administration of probiotics to mothers during pregnancy seen in other studies. Providing probiotics to the mother during pregnancy may face additional challenges related to microbial transfer, as not all infants are exposed to maternal fecal matter or vaginal microbiota in the same manner. Direct probiotic delivery to infants may be more effective in achieving stable colonization due to the specific adaptations of *B. infantis* to the infant gut, as well as the potential for enhanced microbial transmission through breast milk. However, it remains unclear the mechanisms through which certain microbes are trafficked to breast milk or how long supplementation with particular microbiota is needed to induce lasting changes to influence neonatal health.

Due to the aforementioned issues, current recommendations do not generally support routine use of probiotics during pregnancy. However, this does not undermine the importance of optimizing gut microbial composition and diversity during pregnancy for the health of both mother and offspring. Indeed, commercially available probiotic supplements are increasing in number and widely in use without any noticeable concerns. Future studies should be directed towards clarifying optimal strains, dosages, timing, and duration of maternal probiotic administration.

## Conclusion

Maternal intestinal dysbiosis during pregnancy is increasingly recognized as a critical factor influencing offspring microbiome composition and long-term health. Disruptions in microbial communities, driven by factors such as antibiotic use, high-fat diets, and stress, have been associated with adverse outcomes, including metabolic dysfunction, immune dysregulation, and neurodevelopmental disorders in offspring. While preclinical studies suggest potential mechanisms involving microbial metabolites and vertical transmission, significant gaps remain in understanding how maternal microbiota directly contribute to these effects. Probiotic and prebiotic interventions hold promise in mitigating dysbiosis, yet their clinical efficacy remains uncertain due to limited large-scale, longitudinal trials. Future research should focus on elucidating causal relationships between maternal microbiota and offspring health, optimizing microbiome-targeted interventions, and developing strategies to promote a balanced maternal gut microbiome for improved perinatal and neonatal health outcomes.
